# NMN-VD: A Neural Module Network for Visual Dialog

**DOI:** 10.3390/s21030931

**Published:** 2021-01-30

**Authors:** Yeongsu Cho, Incheol Kim

**Affiliations:** Department of Computer Science, Kyonggi University, Suwon 16227, Korea; whdudtn96@kyonggi.ac.kr

**Keywords:** visual dialog, neural module network, attention mechanism, visual coreference resolution

## Abstract

Visual dialog demonstrates several important aspects of multimodal artificial intelligence; however, it is hindered by visual grounding and visual coreference resolution problems. To overcome these problems, we propose the novel neural module network for visual dialog (NMN-VD). NMN-VD is an efficient question-customized modular network model that combines only the modules required for deciding answers after analyzing input questions. In particular, the model includes a *Refer* module that effectively finds the visual area indicated by a pronoun using a reference pool to solve a visual coreference resolution problem, which is an important challenge in visual dialog. In addition, the proposed NMN-VD model includes a method for distinguishing and handling impersonal pronouns that do not require visual coreference resolution from general pronouns. Furthermore, a new *Compare* module that effectively handles comparison questions found in visual dialogs is included in the model, as well as a *Find* module that applies a triple-attention mechanism to solve visual grounding problems between the question and the image. The results of various experiments conducted using a set of large-scale benchmark data verify the efficacy and high performance of our proposed NMN-VD model.

## 1. Introduction

**Figure 1:**
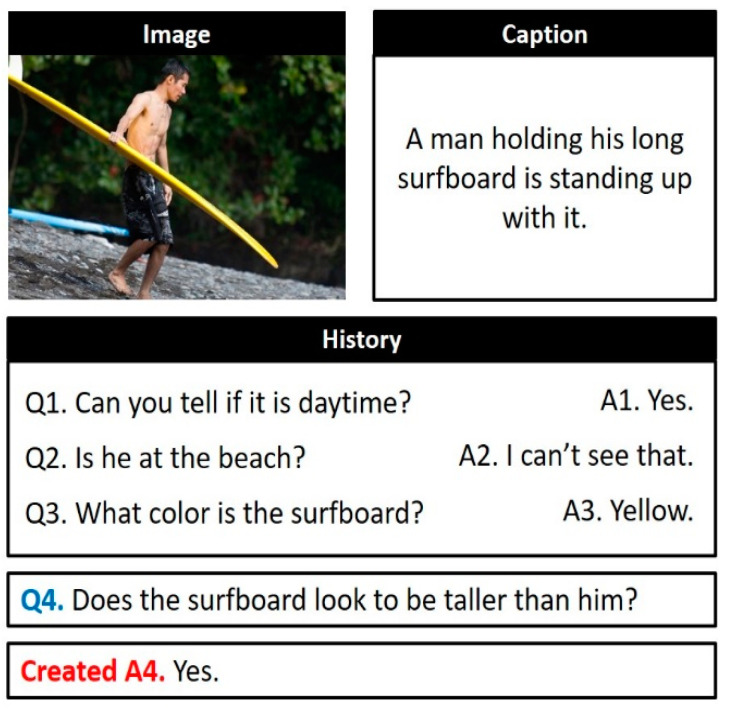
Example of a visual dialog.

Recent developments in computer vision and natural language processing technologies have contributed to increasing interests in multimodal artificial intelligence, which involves the simultaneous understanding of images and language. Representative multimodal artificial intelligence tasks based on images and language include image/video captioning, visual question answering (VQA) [1], and visual dialog [2]. VQA is the task of providing an accurate natural language answer to a natural language question about a given image. By contrast, visual dialog is the task of generating a natural language answer $A_{t}$ to a question $Q_{t}$, given an image and a caption about the image, dialog history about the image $<\left(Q_{1},A_{1}\right),\;\ldots (Q_{t-1},A_{t-1}$)>, and new natural language question $Q_{t}$, as illustrated in Figure 1. As visual dialog extends VQA to a multi-round dialog, it encounters the visual grounding problem, which is a major challenge in VQA.

The visual grounding problem involves identifying the region of the image that the natural language question seeks to understand. For example, in question Q3 in Figure 1, the region of the image indicated by “the surfboard” should be found. Furthermore, as visual dialog exchanges questions and answers consecutively about one image, the question–answer pairs comprising the dialog are interdependent. Owing to this characteristic, visual dialog encounters the visual coreference resolution problem, which involves determining the pronouns and noun phrases in a multi-round natural language dialog that co-refer to the same object instance in an image. For example, to produce the correct answer to question Q4 in Figure 1, it must be known that the pronoun “him” in question Q4, “he” in question Q2, and “man” in caption C refer to the same male region in the image.

The existing models for visual dialog have been mostly implemented with a large monolithic neural network [3,4,5,6,7,8,9,10,11]. However, VQA and visual dialog are composable in nature in that the process of generating an answer to one natural language question can be completed by composing multiple basic neural network modules. Moreover, various types of questions can appear in a visual dialog, each requiring a slightly different detailed process. For example, in the example of Figure 1, question Q3 must find a region corresponding to “the surfboard” in the image and determine the color of “the surfboard” in that region. By contrast, question Q4 must find regions indicated by “the surfboard” and “him” in the image and compare their heights. Thus, it is difficult to learn one monolithic neural network structure that can generate answers to various types of questions. It is likewise difficult for the designer to understand the reasoning process of the neural network via which a large-scale monolithic neural network structure generates answers.

To overcome these problems of monolithic neural network structures, a neural module network (NMN) model for visual dialog [5] was adopted. As natural language questions are inherently compositional, they require multiple levels of reasoning for each question. The NMN model analyzes a question with linguistic substructures and dynamically composes modules on the basis of the structures. Visual grounding is another difficulty encountered; this refers to the understanding of the linguistic elements (e.g., nouns and verbs) that appear in a natural language question in connection with the regions and visual elements in the input image. In addition, visual grounding is highly useful for making a visual dialog model more interpretable because it can explain the rationale for the answer using visual elements. Furthermore, in visual dialog, various comparison questions exist to compare the length, size, color, and other characteristics of two objects in an image. For example, question Q4 in Figure 1 is a comparison question that can be answered only by comparing the heights of “the surfboard” and “him” in the image. Lastly, the visual coreference resolution of pronouns is a major challenge in visual dialog; the impersonal pronouns that appear in dialogs do not need visual coreference resolution, unlike general pronouns. Impersonal pronouns are only used formally, and there are no particular nouns in the dialog history or visual elements in the image that they indicate.

To address these problems, we propose the novel neural module network for visual dialog (NMN-VD), which can lower the complexity of neural network structures and reduce the number of parameters to be learned by selectively composing neural network modules required for the processing of each question. The proposed model has the advantage of more clearly understanding the answer generation process of each question than the monolithic neural network model, and it provides the basic neural network modules for visual dialog: *Find*, *Refer*, *And*, *Relocate*, *Describe*, and *Compare*.

In the proposed model, answers to natural language questions are generated through program generation and execution steps. To solve the visual grounding problem, the proposed model adopts the *Find* module that applies a triple-attention mechanism to improve the find performance, unlike the *Find* modules of existing models that use single- or double-attention mechanisms. Furthermore, to process these comparison questions effectively, the proposed model contains the *Compare* module that determines a minimum bounding area that includes two object regions in the image and extracts a context for comparison operation from this area.

Lastly, the proposed model composes modules to process impersonal pronouns differently from general pronouns. Various experiments were conducted using two large-scale benchmark datasets, VisDial v0.9 and v1.0 [2], to analyze the performance of the proposed NMN-VD.

The remainder of this paper is organized as follows: Section 2 reviews existing work related to this study. Section 3 presents the detailed design of the proposed NMN-VD model. Section 4 describes the implementation of the proposed model and presents experimental results. The conclusions of the study are then presented in Section 5.

## 2. Related Work

### 2.1. Visual Question and Answering (VQA)

VQA [1] is a task in which an appropriate answer is automatically generated to a question when an image and a natural language question about the image are given. To solve the visual grounding problem, which is a major challenge in VQA, early research focused on the attention mechanism. In particular, the visual attention mechanism is a technology that generates a heatmap on the image to indicate candidate image regions from which the answer can be derived. Early studies mainly investigated unidirectional visual attention to find regions related to entities or relationships mentioned in natural language questions [12,13,14,15,16].

Later studies often researched bidirectional attention mechanisms that focus on the relationships between images and natural language questions [17,18,19,20,21]. Lu et al. [17] proposed a model that applied the question attention and visual attention for the first time. Their model was designed to learn the region of the input image and part of the question that should be focused on simultaneously by applying co-attention between the image and natural language questions. Lao et al. [18], Nam et al. [19], and Yu et al. [20] proposed multilevel attention models between image and natural language questions. Furthermore, Yang et al. [21] proposed a model that uses not only the co-attention mechanism between the question and input image but also the question type information.

### 2.2. Visual Dialog

Visual dialog researchers have developed attention mechanisms for images, questions, and previous dialogs to solve the visual grounding problem. Das et al. [2] and Lu et al. [7] adopted a dialog history attention mechanism to find and focus on past dialog history related to the present question. Wu et al. [10], Guo et al. [4], and Yang et al. [11] proposed a model for applying the co-attention mechanism among the three elements of current question, image, and past dialog history to determine the answer to the current question. Gan et al. [3] proposed a model that repeats co-attention among the three elements several times. Idan et al. [22] developed a factor graph-based attention framework, where nodes correspond to utilities and factors model their interactions. Park et al. [23] proposed the multiview attention network (MVAN), which leverages multiple views about heterogeneous inputs on the basis of attention mechanisms.

Unlike the VQA, which is performed once, visual dialog has interdependence between questions and answers because questions and answers are exchanged several times consecutively for one image. Owing to this characteristic, visual dialog has a visual coreference resolution problem. For visual coreference resolution, the visual dialog model of [9] stored pairs of dialog context and its corresponding visual attention map together in an attention memory network for each round of dialog. A dialog context consists of a dialog history and a new question. Furthermore, this model searched for and used a visual attention map of a past dialog context that has the highest relevance in the attention memory network when finding a reference image region of a new question. By contrast, the model proposed by Kottur et al. [5] used a memory network that stores pairs of each noun word appearing in a natural language question or answer during the dialog and the corresponding visual attention region together. In other words, for the visual coreference resolution problem, the models of [5,9] operate a separate attention memory network at the sentence level and word level, respectively. However, the models of [8,6] adopted visual coreference resolution methods that first find the object indicated by a pronoun of the new natural language question in the past history of natural language dialogs before finding the corresponding visual attention map. However, existing studies on visual dialog including [5] have not attempted to process impersonal pronouns separately from general pronouns, unlike the model proposed in this study.

### 2.3. Neural Module Network

In recent times, deep neural networks have shown excellent performance in various computer vision tasks. However, a more complex task leads to a larger size and a more complex structure of the deep neural network. Hence, the network requires several calculation resources and considerable training time to train a large, robust monolithic neural network model. Meanwhile, different computer vision tasks often require several common subtasks. Therefore, when the complexity of tasks is high, the modular approach can be more efficient as it divides tasks into subtasks and composes neural modules appropriate for each subtask [24,25]. Some previous studies applied such NMNs to VQA and visual dialog tasks [5,26,27,28].

Andreas et al. [26] proposed the first NMN model for VQA. This model decomposes a natural language question into linguistic substructures using a semantic parser and generates a customized deep neural network that can provide an answer to the given question by composing the required neural network modules on the basis of these substructures. However, this model has the limitation that a separately developed semantic parser must be used to generate a neural module network that is suitable for the natural language question. Follow-up studies [27,28] proposed an NMN model that can suggest a composition of modules appropriate for the question by itself without using a semantic parser. Kottur et al. [5] proposed the first NMN model for visual dialog. However, the model of [5] cannot distinguish impersonal pronouns from general pronouns. Moreover, it does not contain any independent module to process comparison questions. Thus, the NMN-VD model proposed in this present study can be considered as an improvement to that of [5], as ours separately processes impersonal pronouns, includes a new compare module, and adopts a revised find module.

## 3. Neural Module Network Model

### 3.1. Model Overview

The NMN-VD model proposed in this study selects the most correct answer to the given question by considering the dialog context in the visual dialog. It decomposes a natural language question into language substructures and generates a question-customized program by composing modules appropriate for each substructure. The program is then run to determine the most appropriate answer to the question.

**Figure 2:**
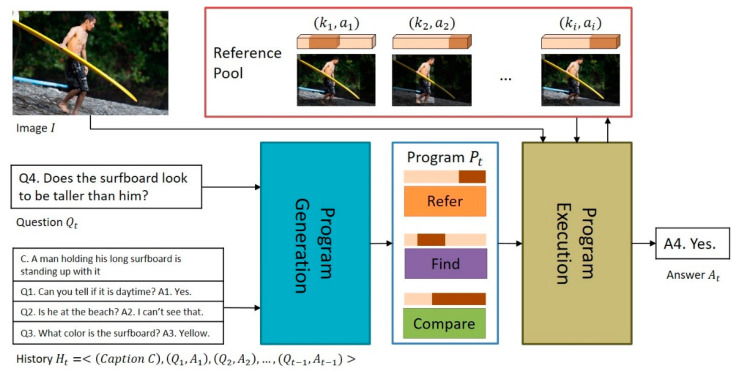
Overall structure of the proposed model.

The structure of the proposed model is shown in Figure 2. The model receives an image I, a dialog history including a short caption C about the image I, $H_{t}=<\left(Caption\;C\right),\left(Q_{1},A_{1}\right),\left(Q_{2},\;A_{2}\right),\ldots ,\;\left(Q_{t-1},A_{t-1}\right)$, and a question of the current round t, $Q_{t}$, as inputs. Then, using these inputs, the proposed NMN-VD model selects the answer $A_{t}$ that is most appropriate for question $Q_{t}$ among the candidate natural language answers of the current round t. The entire process of the proposed model consists of two steps: (a) program generation and (b) program execution. In (a), the program generation step, a question-customized program $P_{t}$ is generated, which includes the modules required for deciding the appropriate answer $A_{t}$ to the question $Q_{t}$ and the execution sequence of the modules. In (b), the program execution step, the context vector $C_{t}$ is generated by dynamically connecting and executing neural network modules according to the generated program $P_{t}$. Then, the most appropriate answer $A_{t}$ is determined in the candidate answer list $L_{t}$ using the generated context vector $C_{t}$. For example, in Figure 2, when the program generator of the proposed model analyzes question $Q_{t}$ using language substructures, the result of [NP (the surfboard), VP (looks to be taller than, NP (PRP(him)))] is obtained, where NP denotes the noun phrase, VP denotes the verb phrase, and PRP denotes the personal pronoun. At this point, the program generator matches the *Find* module to NP, the *Refer* module to the PRP, and the *Compare* module to VP, which contains comparatives such as “taller than”. Consequently, the program generator generates the question-customized program $P_{t}=\left[\mathrm{Refer},\;\mathrm{Find},\;\mathrm{Compare}\right]$ to obtain the answer to the question $Q_{t}$. Next, the program executor of the proposed NMN-VD model determines the most appropriate answer $A_{t}$ in the candidate answer list for the question $Q_{t}$ by connecting and executing the neural network modules according to the generated program $P_{t}$.

**Figure 3:**
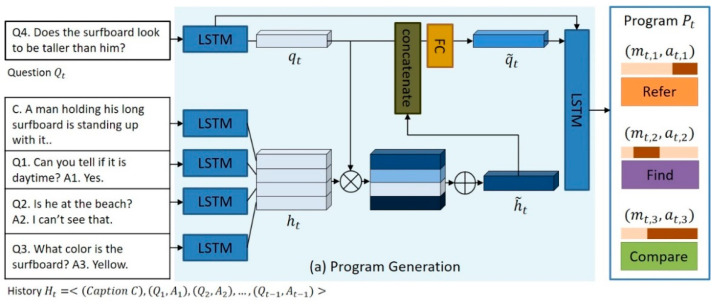
Program generation.

The detailed structure of the program generator is shown in Figure 3. As shown in the figure, the program generator encodes the natural language question $Q_{t}$ as a question feature vector $q_{t}$ through a multilayer long short-term memory (LSTM). Additionally, the program generator generates the dialog history feature vector $h_{t}$ by encoding C and $\left(Q_{i},A_{i}\right)$ constituting the dialog history $H_{t}$ through another multilayer LSTM. Then, the program generator performs an inner product ⊗ operation to calculate the correlation between each question–answer pair $\left(Q_{i},A_{i}\right)$ in the dialog history $H_{t}$ and the current question $Q_{t}$. Furthermore, the program generator generates the final feature vector ${\tilde{h}}_{t}$ of the dialog history $H_{t}$ by calculating the weighted sum ⊕ of the question–answer pairs constituting the dialog history $H_{t}$ on the basis of this correlation. The question feature vector $q_{t}$ and the dialog history feature vector ${\tilde{h}}_{t}$ generated in this way are concatenated, and the question feature vector ${\tilde{q}}_{t}$ considering the dialog history context is generated through the fully connected layer (FC). Lastly, the program generator generates pairs of module labels and textual attention maps $\left(m_{t,i},a_{t,i}\right)$ required to determine the answer to the question $Q_{t}$ one per time through multilayer LSTM as a function of the question feature vector ${\tilde{q}}_{t}$. Consequently, the program generator generates the final output program of the form $P_{t}=$[$\left(m_{t,1},a_{t,1}\right),\;\left(m_{t,2},a_{t,2}\right),\;\ldots ,\;\left(m_{t,i},a_{t,i}\right)$]. In the case of Figure 3, the final program for question $Q_{4}$ is $P_{4}=$[$\left(Refer,a_{4,1}\right),\;\left(Find,a_{4,2}\right),\;\left(Compare,a_{4,3}\right)$], where $m_{4,1}$ of program $P_{4}$ denotes the label “Refer”, which is the neural network module that must be executed first, and $a_{4,1}$ denotes the attention map for “him”, which is a region that must be processed by the Refer module in the natural language question $Q_{4}$. The learning process of the program generator aims to minimize the cross-entropy loss $l_{pg}$ between the program $P_{t}$ predicted by the program generator and the ground-truth program $P_{t}^{*}$.

**Figure 4:**
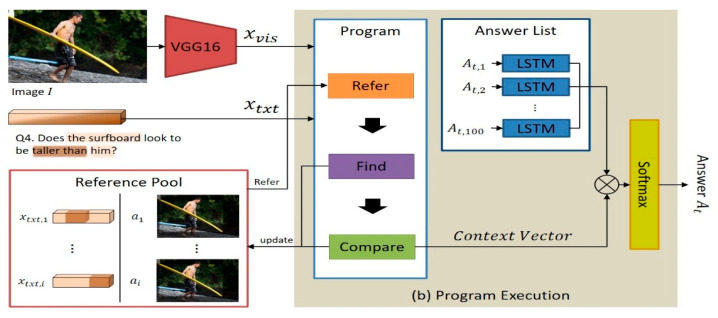
Program execution.

The detailed structure of the program executor is shown in Figure 4. The program executor extracts the visual features $x_{vis}$ from the input image I using the convolutional neural network VGG16. Then, the program executor determines the textual feature $x_{txt}$ of the question region that must be processed by each neural network module $m_{t,i}$ by applying the textual attention map $a_{t,i}$ to the question feature vector $q_{t}$. For most neural network modules, the visual features $x_{vis}$ extracted from the image and the textual features $x_{txt}$ extracted from the natural language question are given as default inputs. Depending on the case, a visual attention map or reference pool is given as an additional input. Once the program executor executes all the neural network modules according to the sequence specified in the program $P_{t}$, the context vector $C_{t}$ to be used for deciding the answer is generated. Subsequently, the program executor calculates the correlation between each candidate answer $A_{t,i}$ (which is encoded through the LSTM) and the context vector $C_{t}$ by conducting an inner product ⊗ operation of both. The probability $Pr_{t}$ of each candidate answer $A_{t,i}$ is calculated on the basis of this correlation. Among the candidate answers, the candidate answer with the highest probability $A_{t,i}$ is selected as the most appropriate predicted answer $A_{t}$. The learning process of the program executor aims to minimize the negative log-likelihood loss $l_{pe}$ between the predicted answer $A_{t}$ and ground-truth answer $A_{t}^{*}$.

### 3.2. Neural Network Module

The list of modules used in the NMN program is provided in Table 1. The modules can be largely divided into two types based on output: the *Find*, *Relocate*, *And*, and *Refer* modules generate an attention map for the input image as an output, whereas the *Describe* and *Compare* modules generate the context vector for deciding the answer as an output. The *Find*, *Refer*, and *Compare* modules are explained in more detail in Section 3.3, Section 3.4 and Section 3.5. The inherent function of each module is described here.

**Figure 5:**
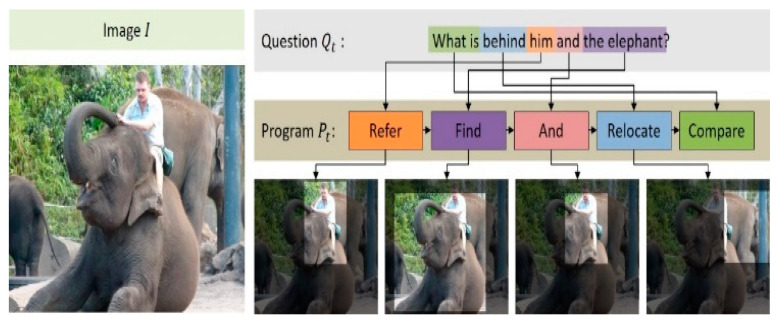
Example of a generated neural program.

As presented in Table 1, the *And* module receives two visual attention maps, $a_{1}\;\mathrm{and}\;a_{2}$, and outputs the image region that is commonly indicated by them. In the example of Figure 5, the visual attention maps corresponding to “him” and “the elephant” are given as inputs of the *And* module; the output of the *And* module here is the common region where objects in the image overlap.

The relocate module performs the function of moving the visual attention map a for the image $x_{vis}$ to the direction indicated by $x_{vis}$. Here, $x_{txt}$ corresponds to a prepositional phrase that appears in the natural language question, such as “behind”, “in front of”, “up”, and “down.” In the example of Figure 5, $x_{txt}$, which indicates the moving direction, corresponds to “behind” that appears in a natural language question. Accordingly, the *Relocate* module moves the common regions of “him” and “the elephant” in this image further back. The operation equation of the *Relocate* module is as follows:

(1) $$y=conv_{2}(conv_{1}\left(x_{vis}\right)⊙W_{1}\left(sum\left(a⊙x_{vis}\right)\right)\;W_{2}x_{txt}),$$

where $x_{vis}$ denotes the visual feature of the image, a denotes the visual attention map of the image, and $x_{txt}$ denotes the natural language question part that indicates a direction.

The *Describe* module plays the role of generating the context vector $C_{t}$, which is required to determine the answer on the basis of the visual attention map a and the natural language question $x_{txt}$ for the image $x_{vis}$. The operation equation for the *Describe* module is as follows:

(2) $$y=W_{3}^{T}\left(W_{1}sum\left(a⊙x_{vis}\right)⊙W_{2}x_{txt}\right).$$

### 3.3. Refer Module

The *Refer* module is a neural network module for solving the visual coreference resolution problem. To find the object indicated by a noun phrase or a pronoun in the question $Q_{t}$, the *Refer* module searches the reference pool. Using a dictionary format composed of key–value pairs, the reference pool stores nouns and noun phrases that appeared in previous questions and answers of dialogs and the image regions corresponding to them. Thus, the key $k_{i}$ in a reference pool is a noun or noun phrase that appears in a question or answer, and the corresponding value is a visual attention map $a_{i}$ indicating the region of the corresponding object in the image. The proposed NMN-VD model finds the visual attention map, which is the region of the corresponding object in the image, using the *Find* module whenever a noun or noun phrase appears in the natural language question of the dialog. At this point, the pair ($x_{txt}$, a) of the noun text $x_{txt}$ (the input of the *Find* module) and the visual attention map a (the output of the *Find* module) is stored in the reference pool.

**Figure 6:**
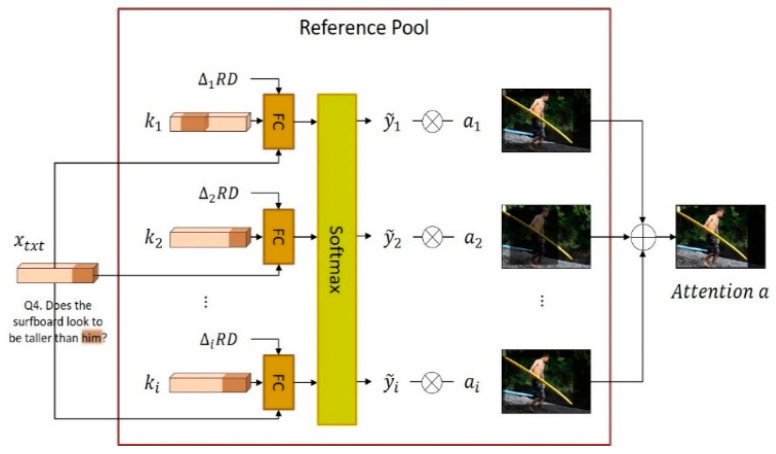
Reference pool and *Refer* module.

Figure 6 illustrates the work of the reference pool and *Refer* module. In this figure, $x_{txt}$ indicates the pronoun “him” of the new question $Q_{t}$, which requires coreference resolution. The *Refer* module of the proposed NMN-VD model searches for the reference key $k_{i}$ that has the highest correlation with the pronoun $x_{txt}$ in the reference pool. The correlation between $x_{txt}$, which is the pronoun part of the question $Q_{t}$, and each key $k_{i}$ of the reference pool is calculated by the scoring network as follows:

(3) $$\tilde{y_{i}}=Softmax\left(W_{1}\left(\left[x_{txt},\;k_{i},\;{△}_{i}RD\right]\right)\right),$$

where ${△}_{i}RD$ denotes the round distance between the current dialog round *t* and the past dialog round in which $k_{i}$ appeared. Considering the sequential nature of dialog, the pronoun of the current question $Q_{t}$ is highly likely to indicate an object that appeared in the most recent dialog round. The scoring network concatenates the pronoun $x_{txt}$, key $k_{i}$ of the reference pool, and dialog round difference ${△}_{i}RD$ between these two into one and evaluates the relevance of each key $k_{i}$ through the softmax layer. As a function of the relevance score $\tilde{y_{i}}$ of each reference key, the equation for obtaining the final attention map a, which is the image region of the target object indicated by the pronoun $x_{txt}$, is as follows:

(4) $$a=\;\sum_{i=1}^{\left|P_{ref}\right|}\tilde{y_{i}}a_{i},$$

where $\left|P_{ref}\right|$ denotes the number of key–value pairs in the reference pool. The relevance score $\tilde{y_{i}}$ of each key $k_{i}$ for the pronoun $x_{txt}$ is used as the weight of the visual attention map $a_{i}$, which is the image region indicated by the key. In other words, the *Refer* module determines the visual attention map a, which is the object region indicated by the pronoun $x_{txt}$, by calculating the weighted sum of the visual attention maps $a_{i}$ stored in the reference pool according to the relevance score $\tilde{y_{i}}$ of each reference key $k_{i}$ for the pronoun $x_{txt}$.

### 3.4. Processing Impersonal Pronouns

We further propose a method of distinguishing between general pronouns that require visual coreference resolution and impersonal pronouns that do not. As mentioned in Section 3.3, in the case of general pronouns, the proposed NMN-VD model solves the visual coreference resolution problem using the reference pool and *Refer* module. However, impersonal pronouns do not require visual coreference resolution because they generally do not refer to specific objects that appear in the previous dialog history. When impersonal pronouns are processed using the reference pool and *Refer* module as in general pronouns, a wrong answer can be derived by connecting the target object regions of the past dialog history unreasonably with impersonal pronouns that are unrelated. Therefore, the proposed NMN-VD model generates a program for impersonal pronouns that is different from the one for general pronouns in the program generation step.

**Figure 7:**
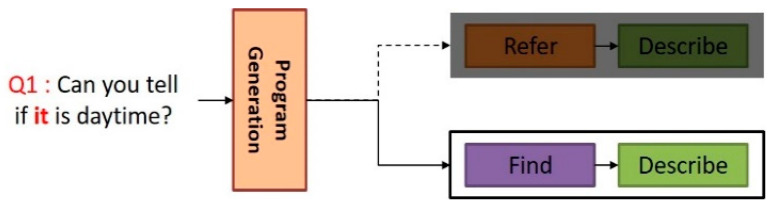
Creating a program for processing impersonal pronouns.

Figure 7 illustrates an example of the program generated by the proposed NMN-VD model to process impersonal pronouns. In question Q1, “Can you tell if it is daytime?”, “it” is an impersonal pronoun and is used to ask about time. When “it” in Q1 is processed as a general pronoun, the generated program will be [*Refer* module, *Describe* module]. However, the proposed NMN-VD model was designed to generate a correct program such as [*Find* module, *Describe* module] by distinguishing “it” as an impersonal pronoun.

### 3.5. Compare Module

This study proposes a new *Compare* module to answer effectively various comparison questions that require comparison of properties (e.g., height and size) between two objects in an image. The inputs of the *Compare* module are the visual attention maps $a_{1}\mathrm{and}\;a_{2}$ of two objects, the visual features of the entire image $x_{vis}$, and the expression of comparison relation $x_{txt}$. Furthermore, the output is the context vector C that indicates the comparison result of the two objects. The *Compare* module requires visual information of the image regions in which the two compared objects are included. Therefore, the *Compare* module in Equation (5) of the proposed NMN-VD model determines the minimum bounding area $\tilde{y}$, which includes the two objects in the image, according to the visual attention maps $a_{1}\;and\;a_{2}$ of two objects, as follows:

(5) $$\tilde{y}=\min\left(Threshold\left(a_{1},a_{2}\right)\right).$$

**Figure 8:**
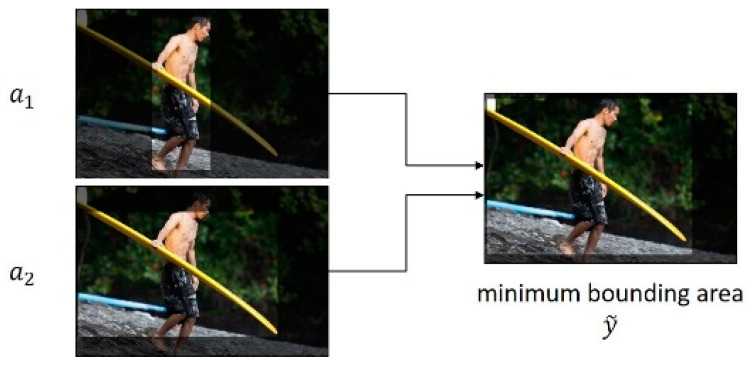
Example of minimum bounding area.

Figure 8 illustrates an example of calculating the minimum bounding areas of two objects for comparison question Q4, “Does the surfboard look to be taller than him?”, which includes the comparison expression “taller than”. In Figure 8, $a_{1}$ and $a_{2}$ denote the visual attention maps of “him” and “the surfboard”, respectively, which are objects of comparison, and $\tilde{y}$ denotes the minimum bounding area of the two objects determined by the *Compare* module.

(6) $$C=\;W_{3}^{T}\left(W_{1}sum\left(\overset{ˇ}{y}⊙x_{vis}\right)⊙W_{2}x_{txt}\right).$$

Furthermore, the *Compare* module generates the context vector C, which is the final result of comparison, as shown in Equation (6), according to the minimum bounding area $\tilde{y}$ of the two objects, visual features $x_{vis}$ of the entire image, and expression of the comparison relationship $x_{txt}$.

### 3.6. Find Module

The proposed NMN-VD model uses the *Find* module to solve the visual grounding problem in a visual dialog. To improve the detection performance, the *Find* module of the NMN-VD model applies a triple-attention mechanism.

**Figure 9:**
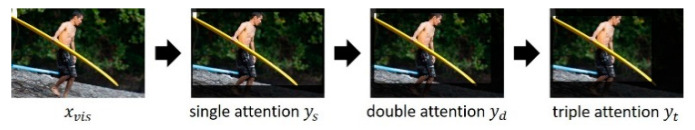
Attention effect.

Figure 9 illustrates the effects of a more accurate detection of the “the surfboard” region in the image with an increasing number of attentions. However, excessive attention above a certain number narrows the detection area, and the correct object region cannot be found. Therefore, the NMN-VD model applies triple attention that detects the object region most accurately.

(7) $$y_{s}=conv_{1}\left(x_{vis}⊙x_{txt}\right).$$

(8) $$y_{d}=conv_{2}\left(\left(\left(sum\left(y_{s}⊙x_{vis}\right)⊙x_{txt}\right)⊙x_{txt}\right)⊙x_{vis}\right).$$

(9) $$y_{t}=\;conv_{3}\left(\left(\left(sum\left(y_{d}⊙x_{vis}\right)⊙x_{txt}\right)⊙x_{txt}\right)⊙x_{vis}\right).$$

Equations (7)–(9) are the equations for obtaining the single-, double-, and triple-attention maps.

## 4. Implementation and Experiments

### 4.1. Datasets and Model Training

We conducted experiments to analyze the performance of the proposed NMN-VD model using VisDial v0.9 and v1.0, which are two benchmark datasets for visual dialog. The VisDial v0.9 dataset is a collection of dialogs that people exchanged about each image through Amazon Mechanical Turk (AMT) on the basis of the MS-COCO image dataset. Each dialog consists of 10 question–answer pairs on average, and 100 candidate answers are given to each question of a dialog. The VisDial v0.9 dataset is composed of training and validation datasets that consist of 82,783 and 40,504 images and dialog data, respectively. VisDial v1.0 is an extension of VisDial v0.9. It has 123,000 images for the training split that combine the training and validation splits of VisDial v0.9. An additional 10,000 images from the Flickr dataset are utilized to construct the validation and test splits in VisDial v1.0, which contain 2000 and 8000 images, respectively. Additionally, ground-truth programs for each question of the dialogs were used for training the program generator of the NMN-VD model in addition to the original VisDial v0.9 and v1.0 datasets.

The proposed NMN-VD model was implemented using the Python deep learning library TensorFlow. Furthermore, the training and performance evaluation experiments of the NMN-VD model were performed in a computer environment with a GeForce GTX 1080 Ti graphics processing unit (GPU) and Ubuntu 16.04 LTS operating system. The model was trained end-to-end, and the total loss for the model training $l_{tot}$ was calculated as follows:

(10) $$l_{tot}=\;l_{pg}+\;l_{pe}.$$

In other words, the total loss $l_{tot}$ is the sum of the losses of the program generation step $l_{pg}$ and program execution step $l_{pe}$. The optimization algorithm used for model training was Adam, and the batch size and epoch were set to 5 and 20, respectively.

### 4.2. Performance Evaluation of Each Module (Ablation Study)

Experiments were conducted to analyze the effect of each module of the proposed NMN-VD model using four different performance metrics. The first performance metric was the mean reciprocal rank (MRR) of the human response in the returned ranked list. MRR is expressed as follows:

(11) $$MRR=\;\frac{1}{\left|Q\right|}\sum_{i=1}^{\left|Q\right|}\frac{1}{rank_{i}},$$

where |Q| denotes the total set of questions, and ${\mathrm{rank}}_{i}$ is the rank of the predicted human answer to the i-th question in the returned ranked list. The second performance metric is Recall@k, which represents the probability of the existence of the human response in the top-k responses to each question. The third performance metric is mean, which is the mean rank of human responses. For example, when the rank of the predicted human responses to five questions is [1, 3, 6, 8, 10], the mean of the ranks is 5.6. The fourth performance metric is normalized discounted cumulative gain (NDCG), which takes into account all relevant answers from the 100-answer list by using the densely annotated relevance scores. It penalizes the lower-ranked answers with high relevance scores. NDCG is computed as follows:

(12) $$DCG@k=\;\overset{k}{\underset{i=1}{\sum }}\frac{relevance_{i}}{log_{2}\left(i+1\right)},$$

(13) $$NDCG@k=\;\frac{DCG@k\;for\;submitted\;raking}{DCG@k\;for\;idel\;raking},$$

where *k* is the number of answer options whose relevance is greater than zero. Of these metrics, a higher score is better for NDCG, MRR, and R@1, 5, 10, but a lower score is better for mean rank.

The first experiment analyzed the effect of attention applied to the *Find* module of the proposed NMN-VD model. The experimental results are summarized in Table 2, where “single attention,” “dual attention,” “triple attention,” and “quad attention” denote the cases of the *Find* module applying one, two, three, and four attentions to the image, respectively.

When the performances of the *Find* module applying single attention, dual attention, and triple attention were compared as shown in Table 2, it was observed that higher numbers of attention to the image improved the performance in almost all metrics. However, when triple attention and quad attention were compared, the case of quad attention, which applied too much attention, showed a lower performance than that of triple attention. Therefore, in the experiment of this study using the VisDial v0.9 dataset, the proposed NMN-VD model demonstrated the best performance when the *Find* module applying triple attention was used. Thus, the *Find* module applying triple attention was used in the remaining experiments.

The second experiment analyzed the *Refer* module (which is used for the coreference resolution of general pronouns in the proposed NMN-VD model) and the effect of the separate processing of impersonal pronouns. To analyze the effect of the separate processing of impersonal pronouns, the VisDialR v0.9 dataset was used in addition to the existing VisDial v0.9 dataset. The VisDial^R^ v0.9 dataset is a partial dataset of dialogs that includes at least one pronoun in the existing VisDial v0.9 dataset. In Table 2, “Refer” indicates the *Refer* module of the CorefNMN model [5], which processes impersonal pronouns in the same way as general pronouns; “Refer + Impersonality” indicates the *Refer* module of the proposed NMN-VD model, which distinguishes general and impersonal pronouns and processes them separately.

As observed in the experimental results in Table 3, for both the VisDial^R^ v0.9 and the VisDial v0.9 datasets, the “Refer + Impersonality” *Refer* module showed a higher performance than the “Refer” *Refer* module. This experimental result confirms that the separate processing of general pronouns (which requires coreference resolution) and impersonal pronouns (which does not) as in the proposed NMN-VD model helped to improve the performance. Furthermore, when the VisDial^R^ v0.9 and VisDial v0.9 datasets were compared, both the “Refer” and “Refer + Impersonality” *Refer* modules showed a higher performance with the VisDial^R^ v0.9 dataset. This result suggests that both *Refer* modules effectively solved the coreference resolution problem of general pronouns.

The third experiment analyzed the performance of the *Compare* module of the proposed NMN-VD model. Table 4 outlines the results of this experiment. In the table, “No Compare” indicates the case of not using a separate *Compare* module, whereas “Compare (Inner Product)” indicates the *Compare* module that performs the comparison operation as shown in Equation (11) [17].

(14) $$y=\;W_{4}^{T}\left(W_{1}sum\left(a_{1}⊙x_{vis}\right)⊙W_{2}sum\left(a_{1}⊙x_{vis}\right)⊙W_{3}x_{txt}\right).$$

The Compare (Inner Product) *Compare* module expressed by Equation (11) applies the attention maps $a_{1}$ and $a_{2}$ for two objects separately to the image without combining them. Furthermore, “Compare (Or)” in Table 4 indicates the *Compare* module that performs a union of attention maps $a_{1}$ and $a_{2}$ of the two objects. Lastly, “Compare (Ours)” indicates the *Compare* module of the proposed NMN-VD model using the minimum bounding area of $a_{1}$ and $a_{2}$.

In the experimental results presented in Table 4, the Compare (Ours) *Compare* module proposed in this study showed the best performance. When the other three modules (excluding Compare (Ours)) were compared, the cases of Compare (Inner Product) and Compare (Or), which added the *Compare* module, showed a slightly lower performance than the case of “No Compare”, which did not include the *Compare* module. The comparison method of Compare (Inner Product) makes it difficult to find the relationship between two objects in an image because they are processed separately rather than in a combined manner. Furthermore, the comparison method of Compare (Or) obtains a distributed visual attention map, although it contains both objects because it uses the union of two visual attention maps. These factors are presumed to be the cause of the lower performance compared with the cases without the *Compare* module.

Contrastingly, the Compare (Ours) *Compare* module proposed in this study uses the minimum bounding area of two objects; thus, it can obtain the information of two objects and their surroundings when answering a comparison question. Therefore, this characteristic of the Compare (Ours) *Compare* module was likely a factor in obtaining a better performance than the other three cases.

### 4.3. Performance Comparison with Existing Models

To prove the efficacy of the NMN-VD model proposed in this study, some experiments were performed to compare the performance with existing visual dialog models. Table 5 outlines the results of the performance comparison experiment with VisDial v0.9 and v1.0. In these experiments, the proposed NMN-VD (Ours) model was compared with the existing models, including late fusion (LF) [2], hierarchical recurrent encoder (HRE) [2], memory network (MN) [2], history-conditioned image attention encoder (HCIAE) [7], attention memory (AMEM) [9], co-attention network (CoAtt) [10], and coreference neural module network (CorefNMN) [5].

As summarized in Table 5, AMEM, CorefNMN, and the proposed NMN-VD model (Ours) demonstrated much higher performances in all metrics in solving the visual coreference resolution problem, compared with the basic models proposed in [2]. This experimental result suggests that solving the visual coreference resolution problem in visual dialog has a significant effect on performance improvement. Furthermore, compared to the monolithic neural network models of [2,7,9,10], CorefNMN and NMN-VD (Ours), which use a modular neural network, showed higher performances. This result means that the question-customized NMN was more effective in processing various question types of visual dialog than a large monolithic neural network.

Lastly, in comparing CorefNMN and NMN-VD (Ours) that use similar reference pools and *Refer* modules for the visual coreference resolution of pronouns, NMN-VD (Ours) showed higher performance improvements in both VisDial v0.9 and v1.0 datasets. Specifically, NMN-VD (Ours) achieved significant improvements in NDCG from 54.79 to 56.90, in MRR from 61.50 to 63.00, in R@1 from 47.55 to 49.23, and in mean from 4.40 to 4.31 for VisDial v1.0. This is because, unlike the conventional CorefNMN model, the proposed model NMN-VD (Ours) includes not only a function to process general and impersonal pronouns separately but also a newly developed *Compare* module for comparison questions and the *Find* module with improved performance. Therefore, the efficacy of the NMN-VD (Ours) proposed in this study compared with existing models was verified through the experimental results presented in Table 5.

### 4.4. Qualitative Evaluation

**Figure 10:**
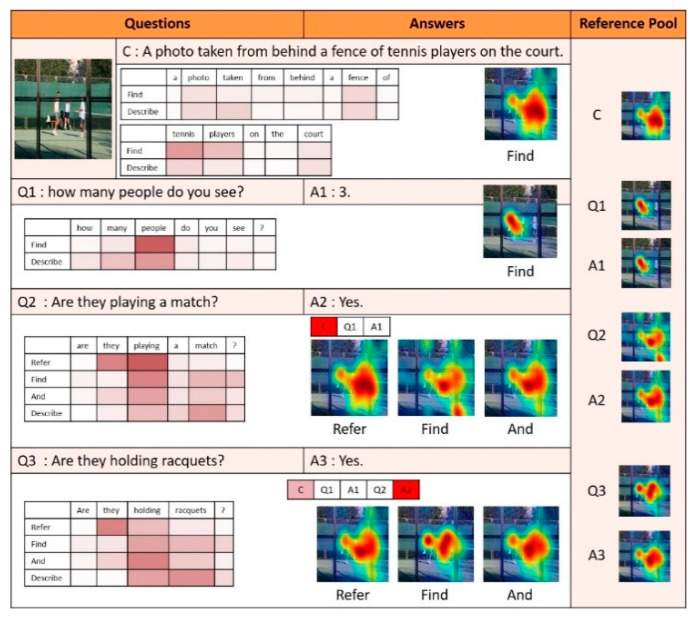
Dialog example 1 derived by the proposed model.

**Figure 11:**
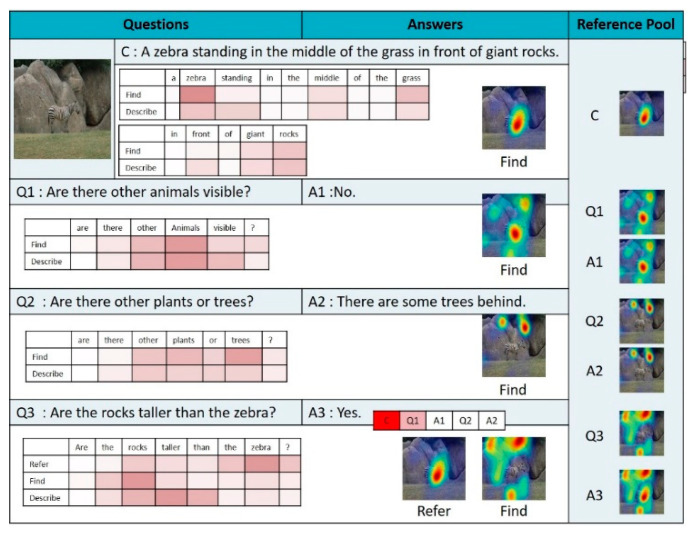
Dialog example 2 derived by the proposed model.

**Figure 12:**
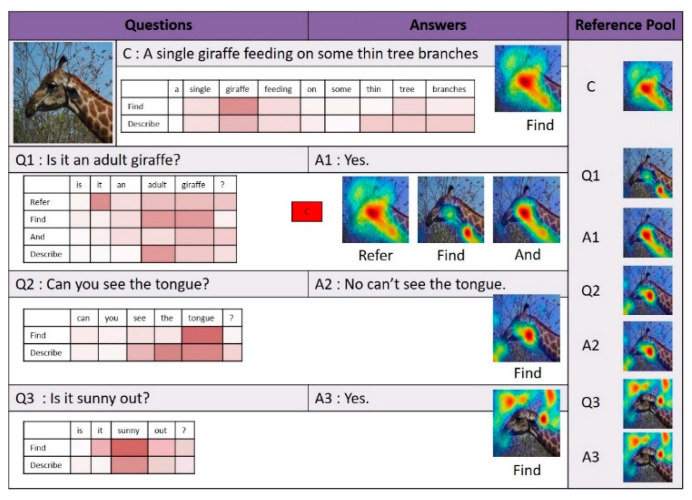
Dialog example 3 derived by the proposed model.

Figure 10, Figure 11 and Figure 12 illustrate examples of visual dialog to which the proposed NMN-VD model was applied. Each figure shows not only the program and predicted answers generated for each question of consecutive questions about one input image but also the changes in the visual attention map, linguistic attention map, and reference pool for each module.

The dialog in Figure 10 illustrates an example of using the *Refer* module of the NMN-VD model. To examine the linguistic and visual attention maps generated first in this dialog, the proposed model detected tennis players and tennis courts mentioned in caption C using the *Find* module and saved them in the reference pool. Then, the model used the *Refer* module for visual reference resolution for the pronoun “they” that appears in questions Q2 and Q3. The pronoun “they” in question Q2 was processed to refer to the same visual attention map as that of the “tennis players” mentioned in caption C. Furthermore, the pronoun “they” in question Q3 was processed to refer to the same visual attention map as that of the previous answer A2.

The dialog in Figure 11 shows an example of using the *Find* module of the NMN-VD model in caption C, question Q1, and question Q2. An examination of the linguistic and visual attention maps reveals that the *Find* module of NMN-VD correctly detected the image region corresponding to the zebra in caption C. Furthermore, it can be seen that the *Find* module of NMN-VD correctly detected the image regions of the corresponding objects required in the answers to the questions about the existence of certain objects in the input image, such as questions Q1 and Q2. Meanwhile, in determining the answer to question Q3 in Figure 11, the *Compare* module of the NMN-VD model was used. To compare the heights of “zebra” and “the rocks”, mentioned in question Q3, the NMN-VD model found the image regions of the corresponding objects in the input image using the *Refer* and *Find* modules. The minimum bounding area of the two objects was then determined using the *Compare* module, and the correct answer to the “taller” comparison relationship was determined as a function of the minimum bounding area.

Question Q3 in Figure 12 illustrates an example of applying the impersonal pronoun processing method of the NMN-VD model. Question Q3 asks about the weather of the input image and contains the impersonal pronoun “it”. The NMN-VD model identified “it” in question Q3 as an impersonal pronoun rather than a general pronoun that requires visual coreference resolution. Therefore, the proposed model detected the sky region required for weather judgment using the *Find* module instead of the *Refer* module and subsequently determined the correct answer. The case analyses of Figure 10, Figure 11 and Figure 12 to which the NMN-VD model was applied confirmed that the *Find*, *Refer*, and *Compare* modules, as well as the impersonal pronoun processing method of the NMN-VD model, were effectively used for various question-answering cases of visual dialog.

## 5. Conclusions

This study proposed a novel neural module network for visual dialog (NMN-VD). It is an efficient question-customized neural module network model that composes only the modules required to determine answers by analyzing the input question. In particular, the model includes a reference module that effectively finds the visual region indicated by pronouns using a reference pool to solve the visual coreference resolution problem, which is an important problem in visual dialog. Furthermore, the proposed NMN-VD model includes a method for processing impersonal pronouns, which do not require visual coreference resolution, separately from general pronouns. Additionally, the model includes a new *Compare* module that effectively processes comparison questions that appear often in visual dialog. Moreover, the proposed model includes the *Find* module, which applies the triple-attention mechanism to solve the visual grounding problem between the question and image. The efficacy of the proposed NMN-VD model was verified through various experiments using two large-scale benchmark datasets, VisDial v0.9 and v1.0.

As a limitation, the current NMN-VD model sometimes composes the same module twice unnecessarily during the program generation process. Therefore, follow-up research is needed to improve the accuracy of program generators. Additionally, the visual dialog dataset VisDial v0.9 contains several unusual questions with low frequencies. Therefore, the NMN-VD model will be extended in a future study to process effectively unusual questions with low frequencies.

## Figures and Tables

**Table 1:** Neural network modules for visual dialog.

Module	Inputs	Output	Function
Find	$x_{vis},\;x_{txt}$	Attention	$y_{s}=conv_{1}\left(x_{vis}⊙x_{txt}\right)$ $y_{d}=conv_{2}\left(\left(\left(sum\left(y_{s}⊙x_{vis}\right)⊙x_{txt}\right)⊙x_{txt}\right)⊙x_{vis}\right)$ $y_{t}=conv_{3}\left(\left(\left(sum\left(y_{d}⊙x_{vis}\right)⊙x_{txt}\right)⊙x_{txt}\right)⊙x_{vis}\right)$
Relocate	$x_{vis},\;x_{txt},\;a\;$	Attention	$y=conv_{2}\left(conv_{1}\left(x_{vis}\right)⊙W_{1}\left(sum\left(a⊙x_{vis}\right)\right)W_{2}x_{txt}\right)$
And	$a_{1},\;a_{2}$	Attention	$y=min\left\{a_{1},a_{2}\right\}$
Refer	$x_{txt},\;P_{ref}$	Attention	${\tilde{y}}_{i}=softmax\left(W_{1}\left(\left[x_{txt},\;k_{i},{△}_{i}RD\right]\right)\right),\;\;\;y=\overset{\left|P_{ref}\right|}{\underset{i=1}{\sum }}{\tilde{y}}_{i}a_{i}$
Describe	$x_{vis},\;x_{txt},\;a$	Context	$y=W_{3}^{T}\left(W_{1}sum\left(a⊙x_{vis}\right)⊙W_{2}x_{txt}\right)$
Compare	$x_{vis},\;x_{txt},\;a_{1},\;a_{2}$	Context	$\tilde{y}=min\left(Threshold\left(a_{1},a_{2}\right)\right)$ $y=W_{3}^{T}\left(W_{1}sum\left(\tilde{y}⊙x_{vis}\right)⊙W_{2}x_{txt}\right)$

**Table 2:** Analysis of the attention effects of the *Find* module. MRR, mean reciprocal rank.

Attention	MRR	R@1	R@5	R@10	Mean
Single attention	64.10	50.92	80.18	88.81	4.53
Dual attention	64.50	51.31	80.52	89.04	4.35
Triple attention	64.70	51.56	80.57	89.07	4.32
Quad attention	64.50	51.41	80.50	88.99	4.35

**Table 3:** Performance comparison between two *Refer* modules.

Dataset	Refer Modules	MRR	R@1	R@5	R@10	Mean
VisDial^R^ v0.9	Refer	64.80	51.64	80.87	89.28	4.29
	Refer + Impersonality	64.90	51.70	80.94	89.35	4.27
VisDial v0.9	Refer	64.70	51.56	80.57	89.07	4.32
	Refer + Impersonality	64.90	51.74	80.88	89.29	4.30

**Table 4:** Performance comparison among *Compare* modules.

Compare Modules	MRR	R@1	R@5	R@10	Mean
No compare	64.90	51.74	80.88	89.29	4.30
Compare (Inner Product)	64.50	51.28	80.50	88.98	4.36
Compare (Or)	64.80	51.67	80.79	89.24	4.30
Compare (Ours)	64.90	51.82	80.88	89.34	4.27

**Table 5:** Performance comparison with existing models.

	VisDial v1.0						VisDial v0.9				
Model	NDCG	MRR	R@1	R@5	R@10	Mean	MRR	R@1	R@5	R@10	Mean
LF [2]	45.31	55.42	40.95	72.45	82.83	5.95	58.07	43.82	74.68	84.07	5.78
HRE [2]	45.46	54.16	39.93	70.45	81.50	6.41	58.68	44.82	74.81	84.36	5.66
MN [2]	47.50	55.49	40.98	72.30	83.30	5.92	59.65	45.55	76.22	85.37	5.46
HCIAE [7]	-	-	-	-	-	-	62.22	48.48	78.75	87.59	4.81
AMEM [9]	-	-	-	-	-	-	62.27	48.53	78.66	87.43	4.86
CoAtt [10]	-	-	-	-	-	-	63.98	50.29	80.18	88.81	4.47
CorefNMN [5]	54.70	61.50	47.55	78.10	88.80	4.40	64.10	50.92	80.18	88.81	4.53
NMN-VD (Ours)	56.90	63.00	49.23	79.81	88.89	4.31	64.90	51.82	80.88	89.34	4.27

LF, late fusion; HRE, hierarchical recurrent encoder; MN, memory network; HCIAE, history-conditioned image attention encoder; AMEM, at-tention memory; CoAtt, co-attention network; CorefNMN, coreference neural module network; NMN-VD, neural module network for visual dialog.

## Data Availability

The datasets used and/or analyzed during the current study are available from the corresponding author on reasonable request.
